# Chronic Renal Disease Complicated by Diarrhea: A Case of Osmotic Demyelination Syndrome

**DOI:** 10.1155/crra/5599298

**Published:** 2026-05-11

**Authors:** Unnati Chouksey, Rahat Brar

**Affiliations:** ^1^ Tata Memorial Hospital, Mumbai, India, tmc.gov.in; ^2^ Homi Bhabha National Institute, Mumbai, India, hbni.ac.in; ^3^ Homi Bhabha Cancer Hospital and Research Centre, New Chandigarh, India

**Keywords:** case report, osmotic demyelination syndrome, pontine and extrapontine myelinolysis, rapid correction of hyponatremia

## Abstract

Osmotic demyelination syndrome is a devastating entity resulting from the body′s inability to accurately respond to a rapid rise in plasma tonicity. In this case report, we describe a 53‐year‐old female patient, a known case of chronic renal failure with superimposed diarrhea developing weakness and confusion over the course of her stay. We aim to discuss and evaluate the role of imaging, specifically the pontine and extrapontine findings and their evolution during the progression and treatment of this condition.

## 1. Introduction

The term osmotic demyelination syndrome (ODS) encompasses two entities, including central pontine myelinolysis in which the pathology is primarily epicentered in the pons and extrapontine myelinolysis involving sites like basal ganglia and cerebral white matter [1, 2]. The crux of ODS lies in rapid correction of hyponatremia, defined as more than an 8 mmol/L increase in serum sodium concentration in a 24‐h period. Apart from this, the initial sodium level before correction, hypovolemia, alcohol use, poor nutritional status, and hypokalemia are other well‐recognized risk factors [1, 3].

## 2. Case Report

A 53‐year‐old female was taken to the emergency department of a local hospital with confusion, dystonia, and seizures for 1 day. She had a known case of hypertension and chronic renal failure for 2 years and was on medical management with antihypertensives and weekly maintenance hemodialysis through a right brachial arteriovenous fistula. Recent history was significant for vomiting and watery diarrhea for 4 days, for which alternative medicines were taken, but to no benefit. The diarrhea was watery, copious, about five times per day, and was not associated with blood or tenesmus.

On examination at the local hospital, there was no motor weakness or slurring of speech. Blood investigations revealed hyponatremia with serum sodium levels of 113 mEq/L and potassium levels of 3 mEq/L. Her hyponatremia was treated with normal saline over 2 days. Her sodium levels after 2 days were 138 mEq/L (as per documentation in the discharge card). Her condition transiently improved over the next 2 days; however, 1 day later, she developed altered behavior and rigidity involving all four limbs. A CT (computed tomography) brain was done which revealed an ill‐defined hypodensity in the pons. Other detailed history about the treatment was not available from the patient.

One week following the onset of the neurological symptoms, she presented to our institute, where, upon admission, she was conscious but disoriented to time, place, and person with rigidity in all four limbs, suggestive of extrapyramidal symptoms with dysphagia and dysarthria, positive Babinski sign, and brisk deep tendon reflexes. There were no sensory deficits or any signs of cranial nerve palsies. Blood pressure was elevated (180/110 mmHg). Blood and CSF samples were sent out to rule out meningitis/encephalitis, which all turned out to be negative. Serum electrolyte examination revealed sodium of 145 mEq/L, potassium of 3 mEq/L, and chloride and bicarbonate levels of 95 and 23 mEq/L, respectively, with euvolemic status. The BUN (blood urea nitrogen) level was 40 mg/dL with a creatinine level of 1.8 mg/dL. LFTs (liver function tests) revealed a serum albumin level of 2.2 mg/dL with no significant rise in the rest of the liver enzymes. Ultrasound abdomen revealed an unremarkable hepatobiliary system with bilateral shrunken kidneys with cortical thinning, consistent with changes of CKD. ECG was unremarkable.

**Figure 1:**
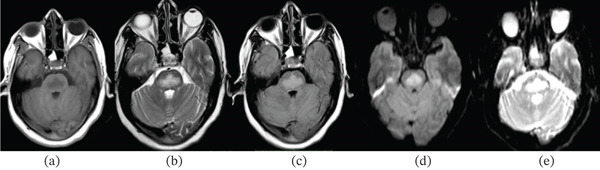
(a–e) T1, T2, FLAIR, DWI, and ADC images of the pontine lesion demonstrating hypointensity on T1, hyperintensity on T2 and FLAIR, and restricted diffusion on DWI images without associated drop on ADC map, demonstrating T2 shine‐through.

**Figure 2:**
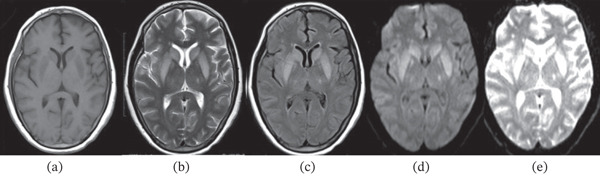
(a–e) T1, T2, FLAIR, DWI, and ADC images of the basal ganglia lesion demonstrating hypointensity on T1, hyperintensity on T2 and FLAIR, and T2 shine‐through.

**Figure 3:**
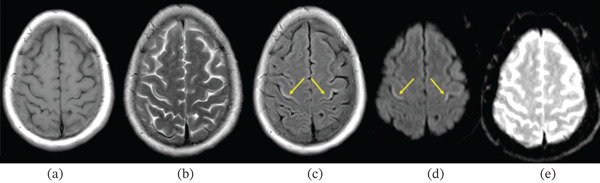
The bilateral precentral hyperintensity is better appreciated on (c, d) FLAIR and DWI images, marked by yellow arrows, and is seen as (a, b) faint T1 hypointensity and T2 hyperintensity, with no associated drop on (e) ADC map. This figure highlights the importance of FLAIR images in identifying this subtle lesion.

MRI brain (performed 8 days after onset of neurological symptoms) revealed T2/FLAIR hyperintensity in the pons and bilateral precentral gyrus with bilateral symmetrical hyperintensities in the caudate nucleus and the putamen. T2 shine‐through (DWI hyperintensity without ADC reduction) was noted in these areas (Figures 1, 2, and 3).

Based on the clinical picture, imaging features, and lab findings, a diagnosis of ODS was proposed, and the patient was shifted to the ICU, with treatment aiming at the correction of the osmotic imbalances while maintaining the eunatremic status. Treatment also included a short course of steroids and levodopa for the extrapyramidal symptoms. During treatment at our institute, the sodium values were gradually corrected to 142, 140, 137, and 135 mEq/L during the 7th, 10th, 15th, and 21st day of admission, and eunatremic status was maintained for the rest of the stay (Table 1).

**Table 1:** Sodium levels of the patient through the treatment course, at the peripheral hospital and at our institute.

	Days since episode	Sodium values
Peripheral institute	1	113 mEq/L
	4	138 mEq/L

Our institute	11	145 mEq/L
	18	142 mEq/L
	21	140 mEq/L
	26	137 mEq/L
	32	135 mEq/L

Over the course of treatment in the hospital, the patient′s general condition and orientation showed rapid improvement over the initial 2 weeks, but the patient showed intermittent spells of aggressive behavior and incoherent speech. The rigidity, dysphagia, and dysarthria however persisted and showed only slight improvement during this period.

Later, the rigidity improved with the patient being able to walk with support; however, there was a lag in improvement while performing hand motor tasks. At discharge, there was residual rigidity involving both hands and dysarthria, with normal memory, behavior, and higher mental functions. Three months following her discharge, mild residual rigidity involving the hands and dysarthria persisted. The patient was advised to get an MRI at this time to look for persisting T2 hyperintensity at the precentral gyrus which would have correlated with the delayed hand motor recovery; however, she did not agree due to financial constraints.

## 3. Discussion

Historically, ODS was thought to be associated with alcoholism and malnutrition; however, it was gradually understood that any condition predisposing to hyponatremia, like severe burns, liver transplantations, anorexia nervosa, hyperemesis gravidarum, and hyperglycemia, rapid correction, can lead to this condition [1,4]. The presenting symptoms generally include dysarthria, dysphagia, dystonia, and flaccid quadriparesis turning into spastic and, if not treated, can be followed by coma and death.

The diagnosis is usually made by matching the imaging findings and the clinical picture, which highlights the importance of eliciting a detailed history. In cases of high index of suspicion in young patients, a toxicology panel should also be included [5].

In a large multicentric study conducted by McMillan et al., ODS was rare despite rapid sodium correction occurring in 17.7% of admissions, and over half of ODS cases occurred without rapid correction, indicating that rapid correction alone is not causative; instead, risk was associated with factors such as very low initial sodium (< 110 mmol/L), alcohol use, hypokalemia, and low urine sodium (≤ 30 mmol/L). Higher desmopressin (DDAVP) use in ODS cases likely reflected a reactive or rescue response to impending or established overcorrection rather than prevention [3].

Imaging findings usually lag behind clinical symptoms; however, restricted diffusion in areas of myelinolysis can be seen as soon as 24 h after symptom onset [1, 4].

ODS occurs due to failure of cerebral adaptive mechanisms during correction of hyponatremia, leading to rapid solute shifts, brain shrinkage, and disruption of tight junctions at the blood–brain barrier, resulting in oligodendrocyte injury and neuronal demyelination. Additionally, downregulation of neutral amino acid transporters during sodium correction impairs cellular amino acid reuptake, further increasing susceptibility to injury. The predisposition to areas like periventricular and subcortical white matter, thalami, basal ganglia, and internal capsule occurs due to the interference of the diffusion of hypertonic edema [6].

CT is generally the first imaging investigation performed at symptom onset when this diagnosis is uncertain; however, CT is much less sensitive than MRI. Areas of myelinolysis are usually hypoattenuating and lack mass effect, usually located within the basilar part of the pons. CT perfusion demonstrates increased blood flow and decreased mean transit time, likely attributing to higher metabolic demands at the site of cell injury [4, 5].

Subtle hypoattenuation, even if present on CT, can be masked by beam hardening artifacts.

**Figure 4:**
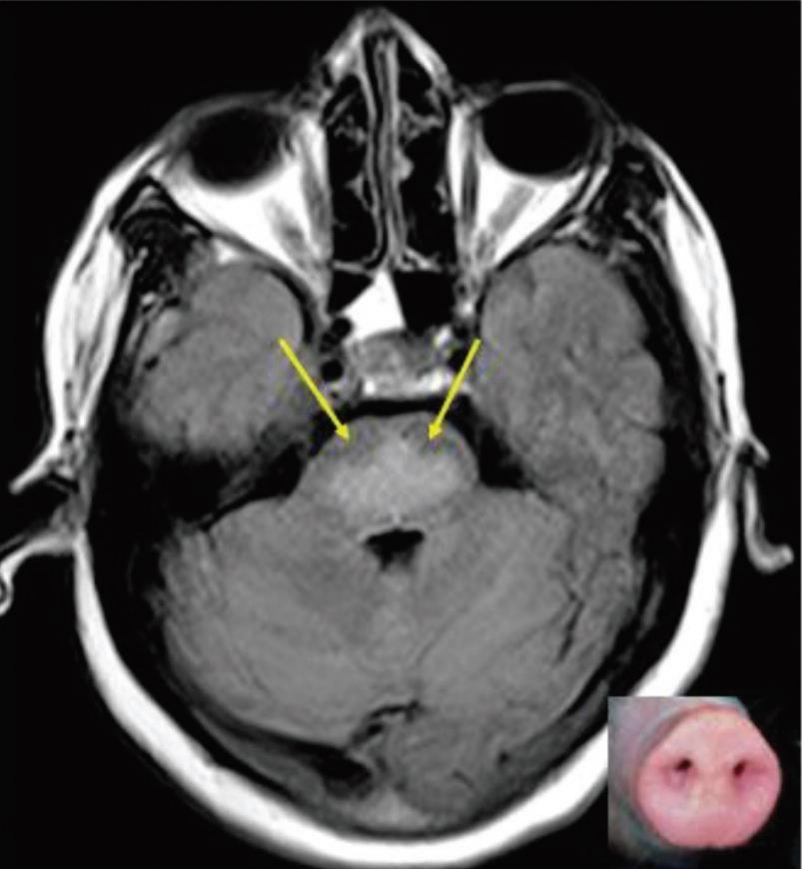
The sparing of the corticospinal tracts in the pontine region (yellow arrows) resembles the snout of a piglet (inset).

On MRI, a symmetric, trident‐shaped T2/FLAIR hyperintensity in the central pons, caused by the relative sparing of the corticospinal tracts, is a characteristic imaging finding. Some authors also refer to this as the piglet sign, referring to the snout of a piglet (Figure 4). Early imaging findings consist of diffusion restriction with associated drop on ADC maps, denoting true restriction, which on temporal evolution of the disease process manifests as T2 shine‐through. They do not show any enhancement on postcontrast imaging. The intensity changes on MRI are thought to be due to a combination of edema and demyelination [5, 7].

The precentral gyrus involvement in this setting is uncommon and is usually associated with amyotrophic lateral sclerosis and fulminant hepatic failure. To the best of our knowledge, there have been two case reports describing the involvement of the precentral gyrus, one following a traumatic brain concussion and the other following thiazide diuretic use for hypertension. There were no other major comorbidities to explain this imaging finding. The cortical laminar necrosis in the precentral gyrus, corresponding to the “hand knob area” as stated in a prior case report, could be due to higher cortical metabolic activity in this area [8]. The delayed improvement of hand motor tasks in this patient could be attributed to the precentral gyrus involvement. A follow‐up MRI would have been beneficial in proving the same.

Symptomatic hyponatremia (< 125 mmol/L) is treated with 3% NaCl boluses aiming for an initial ~5 mmol/L rise in serum sodium, while strictly limiting correction to 8–10 mmol/L in 24 h and using DDAVP if there is a risk of overcorrection or ODS. Once symptoms resolve, hypertonic saline is stopped, and further management is guided by volume status—saline for hypovolemia, water restriction for euvolemia, and diuretics with salt/water restriction for hypervolemia. Asymptomatic chronic hyponatremia does not require urgent treatment and should be managed conservatively with correction of the underlying cause [3, 9].

DDAVP is now increasingly being used as an adjunct in severe hyponatremia to control the rate of sodium correction by limiting free water excretion, a strategy commonly referred to as the “DDAVP clamp.” While this approach helps prevent rapid overcorrection, concerns remain regarding delayed sodium normalization, fluid retention, and prolonged hospitalization. Owing to limited evidence and the absence of clear guidelines on timing, dosing, and patient selection, its use varies widely across clinical settings [9].

## 4. Conclusion

This case underscores the pivotal role of MRI in the timely diagnosis of ODS and in delineating both pontine and extrapontine involvement, including the uncommon finding of precentral gyrus involvement. Recognition of such atypical cortical manifestations may help explain persistent focal motor deficits and broaden the known radiologic spectrum of ODS. Careful clinic radiologic correlation and strict control of sodium correction remain essential to prevent this potentially devastating but avoidable complication.

## Funding

No funding was received for this manuscript.

## Consent

Informed consent has been taken from the patient to publish the details of her symptoms, investigations, and treatment course for the purpose of this case report. No identifiable information is included in the images or clinical data presented in this case report.

## Conflicts of Interest

The authors declare no conflicts of interest.

## Data Availability

Data sharing is not applicable to this article as no datasets were generated or analyzed during the current study.
